# Late-Onset Prosthetic Endocarditis with Paraaortic Abscess Caused by *Cutibacterium acnes*

**DOI:** 10.3390/idr15050059

**Published:** 2023-10-18

**Authors:** Ornela Velollari, Christian Malte Reinhardt, Maike Knorr, Katharina Schnitzler, Dirk Graafen, Matthias Miederer, Ralph Stephan von Bardeleben, Thomas Münzel, Kai-Helge Schmidt, Christian Giebels, Hans-Joachim Schäfers, Lukas Hobohm

**Affiliations:** 1 Department of Cardiology, Cardiology I, University Medical Center Mainz, 55131 Mainz, Germanykatharina.schnitzler@unimedizin-mainz.de (K.S.); tmuenzel@uni-mainz.de (T.M.); kai_helge.schmidt@unimedizin-mainz.de (K.-H.S.); 2 German Center for Cardiovascular Research (DZHK), Partner Site Rhine-Main, 55131 Mainz, Germany; 3 Department of Internal Medicine I, University Medical Center Mainz, 55131 Mainz, Germany; christian.reinhardt@unimedizin-mainz.de; 4 Department of Radiology, University Medical Center Mainz, 55131 Mainz, Germany; dirk.graafen@unimedizin-mainz.de; 5 Department of Nuclear Medicine, University Medical Center Mainz, 55131 Mainz, Germany; 6 Department of Thoracic and Cardiovascular Surgery, Saarland University Medical Center, 66421 Homburg, Germany; christian.giebels@uks.eu (C.G.);; 7 Center for Thrombosis and Hemostasis (CTH), University Medical Center Mainz, 55131 Mainz, Germany

**Keywords:** endocarditis, prosthetic aortic valve, *Cutibacterium acnes*

## Abstract

*Cutibacterium acnes*, an integral component of the skin’s customary bacterial flora, represents a Gram-positive anaerobic bacterium characterized by its low virulence. Despite its low virulence, the pathogen can cause profound-seated infections as well as infections linked to medical devices. We report a case study of a prosthesis endocarditis accompanied by a paraaortic abscess caused by *C. acnes*, a development occurring five years prior to composite aortic root and valve replacement. At the point of admission, the patient presented with a combination of symptoms hinting at a subacute progression, such as weight loss, chest pain, and limitations of cardiopulmonary functionality. An anaerobic pathogen, namely *C. acnes*, was detected in a singular blood culture vial. Since first-line imaging modalities such as echocardiography did not reveal any signs of inflammation, and in the case of a suspected diagnosis for IE, did not show high pretest probability, further diagnostic imaging such as 18F-FDG PET CT was put to use. Here, a highly elevated glucose metabolism around the aortic valve ring was detected, pointing to an inflammatory process. The patient received adjusted intravenous antibiotic therapy over a course of six weeks; he then underwent surgical therapy via re-replacement of the aortic root and valve using a composite conduit. Advanced microbiological analyses, including the amplification of PCR and valve sequencing via 16S rDNA, mainly detected one pathogen: *C. acnes*. Delayed onset with mild symptoms and laboratory findings is characteristic of infective endocarditis by *C. acnes*. Due to its high rate of complications, mortality, and morbidity, an infection should not be disregarded as contamination. Recommendations from different studies underline a combination of a positive blood culture and microbiological evidence to differentiate between contamination and true infection in the case of an infection involving *C. acnes*. Serial blood cultures with prolonged incubation, advanced microbiological analyses, and modified Duke criteria including second-line imaging techniques should be utilized for further evaluation.

## 1. Introduction

Infective endocarditis (IE) is a relatively uncommon yet serious disorder characterized by the infection of native or prosthetic heart valves, endocardial surfaces, or intra-cardiac assist devices. Despite advancements in diagnostic techniques and treatment approaches over the past decade, infective endocarditis remains associated with a considerable incidence of complications and a consequential high case-fatality rate. Notable risk factors for the development of infective endocarditis include prosthetic valve replacements, previous episodes of IE, immunosuppression, venous catheters, intravenous drug usage, and hemodialysis [1,2].

One of the intriguing microbial culprits in infective endocarditis is *C. acnes*, a Gram-positive anaerobic bacterium that normally resides on human skin, particularly in sebaceous gland-rich regions such as the chest, face, and scalp. Although typically recognized for its role in acne, *C. acnes* has low virulence in humans. Certain strains, however, display a propensity for forming biofilms, leading to the colonization of medical devices like prosthetic joints, ventricular shunts, and cardiac implantable devices, consequently giving rise to device-related infections [1,2,3]. Middle-aged men with predisposing factors, including intravascular devices, are notably susceptible to *C. acnes*-related infections. Gender-based disparities are observed, with men exhibiting a higher prevalence of sebaceous glands and therefore *C. acnes* colonization on the chest compared to women [4]. Several factors impact *C. acnes* infections, including biofilm formation’s role in medical device colonization and a reliance on anaerobic conditions that foster tissue infections [5].

Patients with prosthetic valves are at risk of developing infective endocarditis, with reported annual incidence rates ranging from 1% to 6%. This condition’s mortality rates span a wide range, from 21% to 74%. Within the scope of infective endocarditis cases, anaerobic pathogens contribute to 2% to 16% of occurrences. Among these, *C. acnes*, *Bacteroides fragilis*, and *Clostridium species* are the most prevalent. The mortality rate associated with *C. acnes*-related cases is notably high, ranging from 15% to 27%, primarily due to its indolent nature and advanced disease state at the time of diagnosis [6,7,8]. An intriguing observation from a Swedish registry-based study revealed that *C. acnes* contributes to prosthetic valve endocarditis cases in up to 8% of reported instances. Although *C. acnes* IE is not rare, as reported by some case reports and retrospective studies, comprehensive data on its exact incidence, mortality rates, and morbidity remain limited [9,10,11].

In this context, we present a compelling case involving a middle-aged man afflicted with complex late-onset infective endocarditis targeting an aortic valve conduit. The presentation of paraaortic abscess and a subacute clinical course further add to the intricate nature of this case, shedding light on the challenges posed by infective endocarditis and the role of *C. acnes* as an insidious pathogen.

## 2. Detailed Case Description

A 53-year-old male was admitted to our hospital for the evaluation of acute intermittent chest pain. The patient had received an aortic valve conduit (St. Jude Medical Composite 25 mm) five years prior. The troponin levels were serially elevated without presenting ST elevation. A coronary CT angiography presented high-grade stenosis of the proximal left circumflex coronary artery (LCx) and left anterior descending coronary artery (LAD). A coronary angiogram was performed, revealing relevant stenosis of the proximal LCx and LAD; a drug-eluting-stent was inserted into each vessel. An aortography revealed no further conspicuous features. We started with single antiplatelet therapy, clopidogrel, and concomitant long-term oral anticoagulant, phenprocoumon. Despite treatment, the patient suffered from persistent chest pain. A renewed evaluation with a coronary CT angiography demonstrated a hematoma around the aortic root.

**Figure 1:**
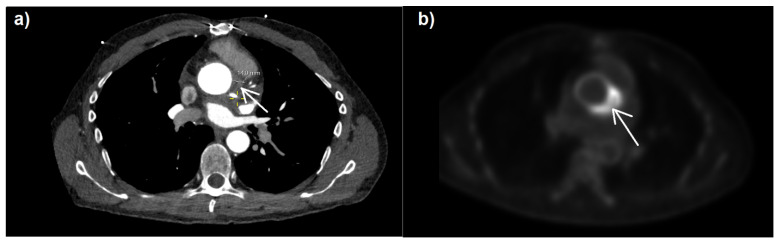
CT (**a**) and ^18^F-FDG PET-CT (**b**) images with attenuated correction in cardiac CT/PET (**b**) and increased FDG uptake (white arrow) around the aortic valve annulus. Department of Nuclear Medicine, University Medical Center Mainz, Germany.

The patient had complained of muscle fatigue and ten kilogram weight loss over the previous six months. There was no elevation of body temperature. The laboratory evaluation demonstrated mildly elevated C-reactive protein levels (17 mg/L; <5 mg/L), with normal levels of procalcitonin (0.02 ng/mL; <0.5 ng/mL) and leukocytes (5.9/nL; [3.5–10]/nL), as well as hemoglobin concentration (12.3 g/dL; [5.5–13, 13–17] g/dL). No petechiae, Janeway lesions, Osler’s nodes, splinter hemorrhages, or Roth spots were observed. A transesophageal echocardiogram revealed no vegetations on the aortic valve. A transthoracic echocardiogram revealed a good left ventricular function (LVEF 55%), a mild aortic regurgitation (Vmax 1.9 m/s, Pmax/mean 15/7 mmHg, AVA 2.3 cm^2^, VTI 15.4 cm), and no regional wall motion abnormalities. The patient had dyspnea with NYHA classification of II, good left ventricular function (LVEF 55%), and no signs of heart failure due to paravalvular leakage of the prosthetic valve. Further inspection through [^18^F]-fluorodeoxyglucose positron emission tomography–computed tomography (^18^F-FDG PET-CT) (Figure 1) revealed highly elevated glucose metabolism around the aortic valve ring and hematoma, indicating a florid inflammatory process.

Empirical antibiotic therapy with vancomycin and gentamycin was started five days after microbiological assessment. During the in-hospital course, blood cultures yielded *C. acnes* from the aerobic vial with a prolonged time-to-positivity of 5 days, 4 h, and 23 min. Repeated blood cultures after a week of antibiotic therapy yielded no growth. The pathogen was resistant to metronidazole and susceptible to clindamycin, imipenem, meropenem, and amoxicillin/clavulanic acid. At this moment, our patient did not fulfill all of the necessary conditions of the modified Duke criteria (one major criterion and two minor criteria were positive, which are listed down below in Table 1) to diagnose infective endocarditis [12]. Similarly, applying the new Duke criteria, one major criterion and two minor criteria were fulfilled, and infective endocarditis by *C. acnes* appeared to be a possible and not a definite diagnosis. Considering the bacteremia with *C. acnes* and signs of inflammatory reaction in the ^18^F-FDG PET-CT, the patient was transferred to the surgical department where the previous operation had been performed. A week after initial admission to our hospital, the patient underwent reoperation; intraoperative findings confirmed the suspected complicated prosthetic valve endocarditis with periaortic abscess and suture line dehiscence to the left ventricular outflow tract, indicating an aneurysm. A repeat Bentall procedure composite valve conduit was performed. 

Histopathological examination of the aortic valve conduit revealed florid, granulomatous inflammation. Microbiological assessment of the tissue sample identified a culture positive for *Staphylococcu saccharolyticus.* The pan-bacterial broad-range 16S rDNA PCR was positive, and subsequent sequencing of the extracted valve material yielded DNA from *C. acnes*. We used MALDI-TOF (Bruker Daltonics Inc., Billerica, MA, USA) mass spectrometry to identify the causative pathogen from the bacterial cultures, further confirming an infection with *C. acnes*. *S. saccharolyticus*, possibly a concomitant pathogen in this case, was resistant to metronidazole and susceptible to vancomycin, clindamycin, meropenem, ampicillin/sulbactam, piperacillin/tazobactam, and penicillin G, therefore displaying a similar resistance and susceptibility profile to *C. acnes*. The clinical condition improved postoperatively. The patient received adjusted intravenous antibiotic therapy with rifampin and ceftriaxone for 6 weeks. In the follow-up, a month after redo surgery, the patient displayed no signs of inflammation, whether clinically or in laboratory findings, as well as no other symptoms. The antibiotic therapy was well received and there were no side effects. Repeated blood cultures with a prolonged incubation period immediately after surgery and at follow-up after one month and three months remained negative and showed no sign of recurrent prosthetic valve endocarditis.

## 3. Discussion and Conclusions

Infective endocarditis resulting from anaerobic bacteria is a relatively infrequent occurrence, accounting for a range of 2% to 16% of cases. Of particular note, *C. acnes*, an anaerobic colonizer within the dermal flora, can play a significant role by serving as the causative pathogen in approximately 8% of documented cases of prosthetic valve endocarditis [4]. Despite its lower prevalence, endocarditis from anaerobic bacteria is associated with a high rate of complications, including congestive heart failure, septic shock, bioprosthetic valve degeneration, and septic emboli, and it has a substantial toll on both mortality and morbidity rates [6,8].

Prosthetic valve endocarditis (PVE) can be temporally classified into early-onset cases, which manifest within the initial 12 months following surgery, and late-onset cases, which emerge beyond this timeframe. In this case, the pathogenic agent caused late-onset PVE of a prosthetic aortic valve with a further complication, e.g., paraaortic abscess. The clinical presentation was characterized by intermittent chest pain, weight loss, fatigue, mild laboratory anomalies, and notably absent fever, indicating a subacute progression. While the presumption is that the source of bacteremia originates from the skin, a possible infection through hematological dissemination in cases of late-onset prosthetic valve endocarditis cannot be ruled out [4]. 

By applying the Duke criteria from the 2015 ESC Guidelines for the management of endocarditis, wherein none of the major criteria were met, the likelihood of the diagnosis of late-onset endocarditis triggered by *C. acnes* did not appear to be likely. In sharp contradistinction, a shift toward the modified Duke criteria, reinforced by prominent, irregular activity proximate to the implantation site as revealed via 18F-FDG PET-CT, heightened the plausibility of prosthesis endocarditis, leading to its ultimate confirmation. Echocardiography, a primary imaging modality for diagnosing prosthetic valve endocarditis, struggles to correctly diagnose IE in up to 30% of cases due to acoustic shadowing limitations [13]. It is unclear whether this phenomenon played a role in not detecting the typical changes pointing to infective endocarditis. Overcoming this diagnostic challenge requires the incorporation of alternative imaging modalities such as 18F-FDG PET-CT, SPECT-CT, or cardiac CT, which effectively elevate the sensitivity of the revised Duke criteria [13]. We endorse the prioritization of the modified Duke criteria, particularly in patients harboring risk factors such as intravascular devices or prosthetic valves, to enhance the probability of promptly diagnosing infective endocarditis and improving patient outcomes. According to the new ESC Guidelines regarding the management of endocarditis, cardiac CT, class I recommendation, plays a pivotal role in the diagnosis of PVE [14]. This is due to its higher accuracy in detecting perivalvular and periprosthetic complications of IE, such as abscess, pseudoaneurysms, and fistulae. Using our case as an example, it becomes obvious that cardiac CT, as an imaging technique, plays a significant role in diagnosing PVE, amongst other things, specifically in the case of acoustic shadowing. In this manner, a comprehensive diagnostic approach, thus integrating clinical information and further imaging modalities, unveils its significance in the management of infective endocarditis. The recommended first-line antibiotic treatment is parenteral penicillin, with or without the addition of aminoglycoside for a minimum period of six weeks. In our case, we decided on dual antibiotic therapy of ceftriaxone and rifampin due to its high risk of complications. There have been numerous studies concerning the efficacy of adding rifampin in treating PVE, due to its ability to penetrate biofilm [15]. In this case, there was only one positive blood culture vial with a prolonged time-to-positivity of more than five days. Repeated blood cultures under antibiotic treatment revealed no positive results. A positive blood culture for *C. acnes* with present symptoms should be cautiously reviewed before being disregarded as contamination. Although time-to-positivity does not help to differentiate between true-positive growth and contaminated growth, at least two blood culture studies with a long time-to-positivity of at least 5 days, if an infection is suspected, should be performed. According to Boman et al. [16], a single positive blood culture for *Cutibacterium* should be considered as sufficient evidence if a foreign intravascular device such as a prosthetic heart valve is present or if the pathogen is isolated at the site of infection. A total of 16% of the positive blood cultures for *C. acnes* in [16] were concluded to be a true infection, thus emphasizing the importance of not disregarding positive findings as contamination, since doing so may miss a true infection.

The new ESC Guideline for the management of endocarditis recommends systematic culture, histological examination, amplification via PCR, and 16S/18S rDNA sequencing in blood culture negative endocarditis [14]. In our case, 16S rDNA PCR was positive, and subsequent sequencing of the extracted valve material yielded DNA from *C. acnes*. It has been reported that valve sequencing is more sensitive than valve culture. In a study by Banzon et al. [17], *C. acnes* was identified in 95% of the reported cases via valve sequencing, which would have been otherwise dismissed as contamination. To differentiate between contamination and true infection, a study by Banzon et al. [17] recommends using at least two criteria to help define a pathogen identified in microbiological testing as the causative agent, such as positive blood culture, positive valve sequencing, positive valve culture, or histopathological demonstrations correlating with such an identified pathogen. In our case report, there was a positive blood culture and positive valve sequencing for *C. acnes*, thus determining such a pathogen as the causative agent for infective endocarditis.

Since anaerobic prosthesis endocarditis is associated with a high rate of complications and mortality, an adequate and prompt diagnosis is crucial to the patient’s outcome. In the 2023 ESC Guidelines for the management of endocarditis, second-line imaging techniques take on a more important role as a class I recommendation in the diagnostic algorithm, if primary imaging modalities such as transthoracic or transesophageal echocardiography provide inconsistent evidence for the suspected diagnosis. To elaborate on the diagnostic pathway, we recommend using a diagnostic strategy consisting of serial blood cultures with prolonged time-to-positivity, extensive microbiological and histological assessment (including PCR amplification and pathogen identification via rDNA sequencing), and further imaging diagnostics such as 18F-FDG PET-CT, cardiac CT, and SPECT-CT.

## Figures and Tables

**Table 1:** Positive modified Duke criteria according to the 2015 European Society of Cardiology guidelines [12].

One Major Criterion	Two Minor Criteria
-Abnormal activity around the site of implantation detected via ^18^F-FDG PET-CT	-Predisposing heart disorder
(only if the prosthesis was implanted for more than 3 months)	-Microbiologic evidence of infection consistent with but not needing major criteria

## Data Availability

The dataset generated and analyzed is not publicly available due to individual privacy concerns, but is available from the corresponding author upon reasonable request.

## Abbreviations

CRP	C-reactive protein
DNA	Deoxyribonucleic acid
ESC	European Society of Cardiology
IE	Infective endocarditis
LAD	Left ascending artery
LCA	Left coronary artery
LCx	Left circumflex artery
PET-CT	Procalcitonin
PET	Positron emission tomography–computed tomography
PVE	Prosthetic valve endocarditis

## References

[1] Achermann Y., Goldstein E.J., Coenye T., Shirtliff M.E. (2014). *Propionibacterium acnes*: From commensal to opportunistic biofilm-associated implant pathogen. Clin. Microbiol. Rev..

[2] Perry A., Lambert P. (2011). *Propionibacterium acnes*: Infection beyond the skin. Expert. Rev. Anti Infect. Ther..

[3] Okuda K.I., Nagahori R., Yamada S., Sugimoto S., Sato C., Sato M., Iwase T., Hashimoto K., Mizunoe Y. (2018). The Composition and Structure of Biofilms Developed by *Propionibacterium acnes* Isolated from Cardiac Pacemaker Devices. Front. Microbiol..

[4] Lindell F., Söderquist B., Sundman K., Olaison L., Källman J. (2018). Prosthetic valve endocarditis caused by *Propionibacterium* species: A national registry-based study of 51 Swedish cases. Eur. J. Clin. Microbiol. Infect. Dis..

[5] Holmberg A., Lood R., Mörgelin M., Söderquist B., Holst E., Collin M., Christensson B., Rasmussen M. (2009). Biofilm formation by *Propionibacterium acnes* is a characteristic of invasive isolates. Clin. Microbiol. Infect..

[6] Brook I. (2008). Infective endocarditis caused by anaerobic bacteria. Arch. Cardiovasc. Dis..

[7] Habib G., Thuny F., Avierinos J.F. (2008). Prosthetic valve endocarditis: Current approach and therapeutic options. Prog. Cardiovasc. Dis..

[8] Lalani T., Chu V.H., Park L.P., Cecchi E., Corey G.R., Durante-Mangoni E., Fowler V.G., Gordon D., Grossi P., Hannan M. (2013). In-hospital and 1-year mortality in patients undergoing early surgery for prosthetic valve endocarditis. JAMA Intern. Med..

[9] Heinen F.J., Arregle F., van den Brink F.S., Marsan N.A., Bernts L., Houthuizen P., Kamp O., Roescher N., Timmermans N., Verkaik N. (2023). Clinical Characteristics and Outcomes of Patients with *Cutibacterium acnes* Endocarditis. JAMA Netw. Open..

[10] Lodhi S.H., Abbasi A., Ahmed T., Chan A. (2020). Acne on the Valve: Two Intriguing Cases of *Cutibacterium acnes* Endocarditis. Cureus.

[11] Patel P.M., Camps N.S., Rivera C.I., Gomez I., Tuda C.D. (2022). *Cutibacterium acnes*: An emerging pathogen in culture negative bacterial prosthetic valve infective endocarditis (IE). IDCases.

[12] Habib G., Lancellotti P., Antunes M.J., Bongiorni M.G., Casalta J.P., Del Zotti F., Dulgheru R., El Khoury G., Erba P.A., Iung B. (2015). 2015 ESC Guidelines for the management of infective endocarditis: The Task Force for the Management of Infective Endocarditis of the European Society of Cardiology (ESC). Endorsed by: European Association for Cardio-Thoracic Surgery (EACTS), the European Association of Nuclear Medicine (EANM). Eur. Heart J..

[13] Scholtens A.M., Budde R.P.J., Lam M.G.E.H., Verberne H.J. (2017). FDG PET/CT in prosthetic heart valve endocarditis: There is no need to wait. J. Nucl. Cardiol..

[14] Delgado V., Ajmone Marsan N., de Waha S., Bonaros N., Brida M., Burri H., Caselli S., Doenst T., Ederhy S., Erba P. (2023). 2023 ESC Guidelines for the management of endocarditis. Eur. Heart J..

[15] Charles P., Hot A., Ou P., Carbonnelle E., Sidi D., Nassif X., Lortholary O. (2007). *Propionibacterium acnes* endocarditis in an adolescent boy suffering from a congenital cardiopathy. Pediatr. Infect Dis. J..

[16] Boman J., Nilson B., Sunnerhagen T., Rasmussen M. (2022). True infection or contamination in patients with positive *Cutibacterium* blood cultures—A retrospective cohort study. Eur. J. Clin. Microbiol. Infect. Dis..

[17] Banzon J.M., Rehm S.J., Gordon S.M., Hussain S.T., Pettersson G.B., Shrestha N.K. (2017). *Propionibacterium acnes* endocarditis: A case series. Clin. Microbiol. Infect..

